# Epidemic Erysipelas

**Published:** 1836

**Authors:** Jesse Paramore

**Affiliations:** Eaton, Ohio


### WESTERN JOURNAL

OF THE

### MEDICAL AND PHYSICAL SCIENCES:

**For October, November, and December, 1836.**

### ESSAYS AND CASES.

### Art. I.—Epidemic Erysipelas.

*Cases of Epidemic Erysipelas*, by Dr. Jesse Paramore, of Eaton, Ohio.

During the fall and winter of 1835, and early part of the spring of 1836, a portion of this county, (Preble) was visited by epidemic erysipelas, which prevailed to a considerable extent until about the middle of February, when it abated, but did not entirely disappear until some weeks afterwards. There were, however, but few cases that terminated fatally, none indeed, that came within my immediate knowledge, except where the disease attacked persons whose constitutions were broken down by old age or by previous disease. The only case seen by me which came to a fatal termination was that of a lady, whom, as I was informed had recently suffered severely from a tedious and painful *accouchment*, followed by a violent attack of Phlegmasia Dolens, and when she was evidently recovering from this, but in a very feeble condition, was violently attacked with erysipelas, which spread over the abdomen generally, and terminated fatally in a few days.

Having learned that this disease, at the time above mentioned, prevailed to some considerable extent in the western country, and that some diversity of opinion existed among the practitioners of the Miami Valley, as to the proper method of treatment I have taken the liberty of transmitting, for the Western Journal the following sketch, taken from my notes of a few of the cases which came under my notice; they will serve as specimens of the whole.

Case. 1st. A small boy aged about three years was on the 7th December attacked with a severe pain in the left knee, which was followed in a few hours by violent convulsions. On the 8th I was called to see him, found the leg much swolen from the knee to the hip, bearing all the marks of erysipelas, which extended over the scrotum; fever considerable, with occasional convulsions. I immediately took 12 oz. of blood, gave him an emetic, and ordered it to be followed by a mercurial cathartic. 9th. Carthartic had operated well, convulsions subsided, fever diminished. Cathartic repeated, ordered an external application of a solution of the acet. plumbi. 10th. Symptoms more favorable, swelling diminished, less pain, and but little fever, cathartic repeated and the external application continued. 11th. Dismissed cured, since which time he has continued to enjoy uninterrupted health.

Case. 2d. Mrs. M., aged 28 years, was on the 13th December, seized with violent rigors, followed by high fever and delirium, on the 14th I was called to see her, when I drew about 40 oz. blood, gave an emetic, and ordered it to be followed by a mercurial cathartic. 15. Emetic and cathartic had operated well, fever less violent, no delirium, but some erysipelatus swelling on and about the nose, with pain in the back and head, cathartic repeated. 16th. Recurrence of chill followed by violent fever and delirium, erysipelatus swelling much increased, extending over the face and head, took 30 oz. blood, and ordered a repetition of a cathartic. 17. Swelling of the face, and delirium increased, took 30 oz. blood, and ordered 20 grs. calomel every four hours. 18th. Symptoms same as yesterday, swelling increased, eyes entirely closed, took 20 oz. blood, calomel continued as before. 20th. Calomel operated well, fever and delirium much abated, partial perspiration, ordered pulv. doveri every two hours. 21st. 3d dose pulv. dor. produced general perspiration, less fever, mind tranquil, swelling much diminished, ordered sulph. quinine, 2 gr. every 3 hours. 23d. Fever gone, no delirium, swelling diminished, quinine continued every four hours, with cathartic. 24. Improving rapidly, prescription same as yesterday, only continued at longer intervals. 29th. Dismissed cured.

Case 3d. Mr. Q., aged about 65, was on the 18th December taken with a burning pain in one of his fingers of the left hand, on which a small blister soon appeared, the hand became much swolen, and the pain extended to the shoulder, severe rigors were experienced followed by flushes of fever, and during the night his mind was much disturbed. No medical aid was called until the evening of the 22d, when I first saw him, the swelling had then extended from the hand to the shoulder, implicating the whole arm, and was of a dark red color, dotted over with many small blisters, pulse 120 in a minute, a great heat with a dry tongue and fauces, and so completely comatose, that it was almost impossible to rouse him. I at once took 40 oz. of blood from a large orifice, which very much relieved the coma, after which I ordered an active emetic, cathartic, and the external application of acet. plumb. 24th. Less pain, but little fever, swelling much diminished, external application continued, gave 20 grs. calomel which was followed in three hours by ol. ricini. and spts. turpentine. 25th. Dismissed cured.

Case 4th. H. E. aged 60 years, was on the 13th December attacked with a violent pain about midway between the elbow and shoulder of the left arm, accompanied with rigors, which were soon followed by violent fever and delirium, the limb soon became very much swolen, and in a few hours some vesication was apparent. No medical aid was had until the 25th of the same month, at which time I was called to see him, when I found the whole skin and cellular substance of the arm from the hand to the shoulder, gangrenous, except a strip on the inner side, about one and a half inches in width. The lines between the sound and diseased parts being distinctly marked, I traced them with a pair of curved scissors, and removed a mass of mortified skin and cellular substance together with the fascia of the muscles without pain, divesting the whole arm of any covering except the narrow strip above mentioned. The muscles appeared quite sound, and in a tolerably healthy condition, I then covered the limb with pulverized charcoal, and ordered like dressings, renewed every twelve hours, and laxatives to keep the bowels open with a liberal use of stimulants and tonics, under which treatment he improved rapidly. When granulations began to form, the dressings were basilicon, and on the 23d January, the limb was entirely healed. And what is to me somewhat surprising, he has regained the perfect use of the arm, except a partial stiffness of the elbow joint, and that, bids fair to regain its former usefulness.

*Eaton, Ohio, August 24th*, 1836.

